# The Metric System

**Published:** 1876-10

**Authors:** M. S. Bidwell


### THE METRIC SYSTEM.

M. S. BIDWELL.

This system commends itself to thinking people by its scientific basis, by its uniformly decimal character, so convenient for calculation, and above all, by the simple relation of its different parts. For common purposes of trade, a binary division will always be preferred, but for calculation, and as a basis of this, a decimal plan must sooner or later be adopted, and the metric system will be the one. Already adopted by more than half the governments of the civilized world, legalized in Great Britain and the United States, and universally used by scientific men, its adoption by all nations would seem to be only a question of time. Even now, in order to understand any European scientific work, some acquaintance with the metric system is indispensable. And yet this knowledge seems almost entirely wanting among Americans. For instance, would it not be entirely safe to say that of our physicians (selecting them not invidiously, but as an intelligent and educated class of men, who could use this knowledge to advantage), at least nine-tenths have no distinct conception of the metric measures. To them a litre, a centimetre, or a gramme, has no meaning and conveys no definite idea. The reason of this general ignorance, or at least one reason, is evident. The student inquiring about the metric system, or referring to the tables in which it is set forth, is confused and discouraged by the array of unfamiliar names, thirty or forty in number, most of them four or five syllables long, with their equivalents in our measures carried out to three or four places of decimals. Perplexed by Millilitres and Myrialitres, Decigrammes and Decagrammes, it is no wonder that he closes the book in despair, thinking (as a very intelligent young physician once said to the writer), "That is too deep for me?" But the fact is that you do not need to learn all these thirty or forty names, nor their exact mathematical value. There are only six, or eight at the most, that are used at all, or at least that are likely to be met with in books, and all these are readily reducible, approximately, to our measures. The six essential measures are, of length, the Metre, Centimetre, and Millimetre; of capacity, the Litre and Cubic Centimetre; and of weight, the Gramme.

The Metre, the basis of the whole system, is about 40 inches; more exactly, it is 3 feet, 3 inches, and 3 eighths of an inch—easily remembered as three threes. For subdivision, it is convenient to consider it 40 inches. The Centimetre is therefore about 2-5 of an inch, and the Millimetre 1-25.

The Litre is a cubic Decimetre; a box 4 inches each way represents it pretty well. It is just a quart, being intermediate between our wine and dry quarts. Small quantities are measured by Cubic Centimetres (cc.) of about 16 minims, or 1-30 of a fluid ounce.

The Gramme (gram) is the weight of a cubic centimetre of water; it is equal to 15.43 grains, or about 1-30 of an ounce. Smaller weights are usually expressed in decimals of a gramme.

These six comprise all the metric measures commonly used in scientific works. To them may be added, if you choose, the Kilogram, about two pounds avoirdupois, being the weight of a litre of water, and the Kilometre, the road measure, which is about 5-8 of a mile. It is not generally known that our present nickel five cent piece is a metric measure, being exactly 2 centimetres in diameter aud 5 grammes in weight.

Minute accuracy has not been aimed at in this article so much as practical utility and convenience of recollection. If it shall aid in stripping this very important system of some of its imaginary terrors, and making it more of a reality to any reader af the BISTOURY, the writer's purpose will be accomplished.

